# Class III Restoration of Anterior Primary Teeth: *In Vitro* Retention Comparison of Conventional, Modified and Air-abrasion Treated Preparations

**DOI:** 10.5681/joddd.2014.016

**Published:** 2014-06-11

**Authors:** Naser Asl Aminabadi, Ebrahim Najafpour, Leila Erfanparast, Mohammad Samiei, Monireh Haghifar, Alireza Sighari Deljavan, Zahra Jamali, Fatemeh Pournaghi Azar, Marzieh Shokravi

**Affiliations:** ^1^Professor, Department of Pediatric Dentistry, Faculty of Dentistry, Tabriz University of Medical Science, Tabriz, Iran; ^2^Assistant Professor, Department of Paediatric Dentistry, Faculty of Dentistry, Tabriz University of Medical Science, Tabriz, Iran; ^3^Assistant Professor, Department of Endodontic, Faculty of Dentistry, Tabriz University of Medical Science, Tabriz, Iran; ^4^Post-graduate Student, Department of Paediatric Dentistry, Faculty of Dentistry, Tabriz University of Medical Science, Tabriz, Iran; ^5^Research Assistant, Faculty of Dentistry, Tabriz University of Medical Science, Tabriz, Iran; ^6^Assistant Professor, Department of Oral Science, Faculty of Dentistry, Tabriz University of Medical Science, Tabriz, Iran; ^7^Assistant Professor, Department of Operative Dentistry, Faculty of Dentistry, Tabriz University of Medical Science, Tabriz, Iran

**Keywords:** Anterior primary teeth, air abrasion, composite restoration, surface treatment

## Abstract

***Background and aims.*** Anterior esthetic restoration is challenging in pediatric dentistry, due to limited durability and poor retention of the restoration.This study assessed the effect of air abrasion on tensile failure load of composite class III restorations using different preparation techniques.

***Materials and methods.*** 100 extracted human anterior primary teeth were divided, based on the preparation methods, into four groups each consisting of 25 subjects : conventional (A), labial surface bevel (B), conventional + air abrasion (C), and labial surface bevel + air abrasion (D). After restoring cavities, tensile failure load of samples was measured in Newton by Universal testing machine at a crosshead speed of 1 mm per minute. The data were analyzed by Kruskal-Wallis and Mann Whitney U tests using SPSS software.

***Results.*** There were statistically significant differences between groups A and C (P = 0.003), groups A and B (P & 0.001), groups A and D (P & 0.001), groups B and C (P = 0.028), groups B and D (P = 0.027), and also groups C and D (P& 0.001). Group D demonstrated the highest mean tensile failure load.

***Conclusion.*** Labial surface bevel treated by air abrasion showed significantly more retention of composite restoration.

## Introduction

The esthetic restoration of anterior primary teeth has long been a challenge in pediatric dentistry. The anterior primary teeth have shown less retention of restorative material compared to the permanent teeth because of the small size of the teeth, close proximity of pulp to tooth surface, relatively thin enamel and surface area for bonding, issues related to child behavior and finally cost of the treatment.^1,2^ In addition, lower bond strength in primary teeth is attributed to a less mineralized dentin, thicker hybrid layer that is not completely penetrated by the bonding agent, different microcrystal arrangement, and the prismless layer that does not respond well to acid etching.^3,4^ Conventional treatment modalities range from fluoride gel to complete-coverage stainless steel crowns; however, the most durable restorations remain the least esthetic.1 Therefore, many revolutionary techniques and materials including the enamel’s prismless layer removal before acid etching and mechanical locks or slots have been suggested to increase surface area for acid etching and bonding to overcome these barriers.^5^

Recently, preparing the entire facial surface and veneering the surface for additional bonding has been proposed to increase the surface area of the enamel for etching and improve retention of class III restorations in primary teeth.^6^ On the other hand, it has been shown that application of airabrasion increases the shear bond strength of composite to enamel and dentin by producing a rough irregular surface and increased surface area. In addition, airborne-particle abrasion increases the wettability of tooth structure, providing additional mechanical retention to the adhesive system, and enhancing the effectiveness of the dentin adhesive system.^7,8^ As a result, significant differences in the adhesive strength after acid etching and air abrasion between primary and permanent teeth have been reported.^9,10^ In a similar context, air abrasion combined with acid etching appears to provide the best conditions for enamel treatment prior to sealant placement.^11^ Moreover, highest tensile strength of composite resin to enamel was obtained with air abrasion followed by acid etching.^12^

In the light of these reflections, we aimed to assess the effect of different preparation techniques including conventional, conventional + air abrasion, labial surface bevel and labial surface bevel + air abrasion preparations in class III composite restorations of primary teeth on tensile failure load. Thus, two sets of variables, including (A) type of cavity preparation with and without air abrasion; and (B) tensile failure load were analyzed to answer the following research question: How different preparation techniques with and without air abrasion treatment influence tensile failure load of class III composite restorations in primary teeth? It was hypothesized that different preparation techniques with and without air abrasion treatment could affect differently tensile failure load of class III composite restorations in primary teeth.

## Materials and Methods

### Tooth Selection 

For this in vitro study, which was approved by the Ethic Committees of Tabriz University of Medical Sciences (Ref number: 7648), one hundred extracted human primary incisor teeth were obtained from the children in the Department of Pediatric Dentistry, with at least one proximal surface free of caries and enamel malformation. Deposits and soft tissue residues were carefully removed from tooth surfaces using rubber cup and water-pumice slurry.

###  Sample Size and Grouping 

According to the pilot study, considering α = 0.05, power = 80% and difference 5 Newton of failure load in the conventional + air abrasion and labial surface bevel + air abrasion group (main groups), 22 samples for each group and thus a total sample size of 88 estimated in the study. For increasing the validity of study, 100 samples were selected and randomly divided into four groups of each 25 according to the cavity preparation method and conditioning approaches as follows: Group A, conventional preparation; Group B, labial surface bevel; Group C, conventional preparation + air abrasion treated; and Group D, labial surface bevel + air abrasion treated.

### Specimen Preparation 

All the teeth were stored in 0.5% chloramine-T (Formula & Acao, Sao Paulo, Brazil) solution. Teeth were then mounted two millimeters below the CEJ, approximately at the level of the alveolar bone in a healthy tooth in self-cured acrylic resin in cylindrical plastic molds while making the labial tooth surface parallel to the walls of a plastic mold.

###  Treatments

The cavity preparations were standardized according to the established protocols including incisogingival dimension of 2 mm, the cavity depth 1 mm and buccolingual dimension 2 mm.^13^ The attempt was made to allow the same thickness of tooth structure within each group and thickness of cavity walls were standardized with the aid of an orthometer gauge (KorkhausOrthometer Kit, 75228 Ispringen, Dentaurm, Germany). A high-speed water spray bur was used for each preparation. Class III cavities was prepared in each tooth using 008 diamond fissure bur (D&Z, Wisbaden, Germany) with a high speed hand piece under water spray. The diamond burs were replaced every five cavity preparations and the air abrasion instrument was cleaned after any two applications.^14^

In group A, conventional Cl III cavity preparation in either mesial or distal surface of teeth was performed. In group B, a modified Cl III cavity was prepared with 0.5 mm labial bevel from mesio-labial or disto-labial line angle to the other side.^13^ In group C, a conventional Cl III cavity was prepared as described for group A and the subjects in group D was treated with a modified Cl III cavity as described for group B. In groups C and D, the prepared surfaces were rinsed and dried and then treated by an air-abrasive system (Dental Microblaster, MicroblasterDento-Prep, Denmark) using 50 µm aluminium oxide particles stream perpendicularly to the surface at 80 psi air pressure for 15 seconds. The treatments were accomplished at a distance of approximately 5 mm from the cavity surfaces.^10^ Extra-oral evacuation system was used to remove dry particles. Airborne-particle–abraded specimens were thoroughly rinsed with vigorous water spray for 30 seconds to clean the surfaces from residual alumina particles.

After completion of the cavity preparations, all subjects received surface treatment with a 35% phosphoric acid gel (N-Etch, Ivoclar Vivadent, Schaan, Liechtenstein) that applied to enamel and dentin with light scrubbing motion for 30 seconds. The cavities were rinsed with air/water spray for 20 seconds and gently dried with air to keep the tooth surface moist. Then, One-Step Plus adhesive system (Single Bond, 3M ESPE, St. Paul, USA) was applied by two consecutive coats, with a clean microbrush (Microbrush Co., Greyton, USA) and gently blot-dried for 5 seconds to evaporate the solvent and was polymerized for 20 seconds using a visible light-curing unit (Ivoclar Vivadent, Schaan, Liechtenstein) with an output of 400 mW/cm2.^13,14^

**Figure 1.**
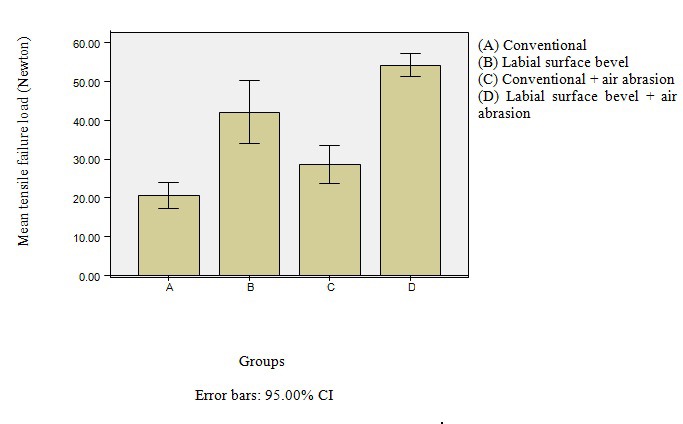
Tensile failure load of Cl III composite restorations in anterior primary teeth in the studied groups.

Then, composite (3M, Dental Products, USA) was applied and cured for 40 seconds.^15^ A transparent matrix bond was placed on the last layer to remove the material excess and complete the setting process. A 0.5 mm round stainless steel wire with 5 cm length was embedded in the composite material at angle perpendicular to the proximal surface of the teeth. The composite resin was applied to cover the retainer surface and photo-polymerized for 40 seconds on each tooth surface (Figure 1). After completion of the restorations, the specimens were polished with diamond polishing burs (D&Z, Wisbaden, Germany) and polishing disks (Sof-LexTM, 3M ESPE, St.Paul, USA) under simultaneous water cooling.^16^

###  Failure Load Assessment 

To simulate oral cavity conditions, a thermocycling procedure using thermo-cycling machine was undertaken, which consisted of 500 cycles at 6°C and 60°C, with a dual time of 30 second each.^16,17^ Specimens were stored in distilled water at room temperature until all samples were ready for tensile failure load testing. The retention force was tested by Universal testing machine (H5k-S, Hounsfield Test Equipment, UK). The wire was grasped with machine jigs and standard load was applied via the wire to the restoration at a head speed of 1 mm/minute until restoration failure.^18^ Failure load (in Newton) was the restoration’s retention force (Figure 1).

### Statistical Analysis 

All data was presented as Mean ± Standard deviation. The main statistical assessments addressing the research question were Kruskal-Wallis test and Mann-Whitney U test to compare the data. Data were analyzed using SPSS software (version 16). P < 0.05 was considered statistically significant.

## Results

**Figure 2.**
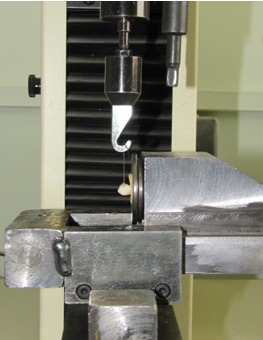
Universal testing machine and tensile failure load test.

The average tensile failure load (Mean ± SD) was 20.66 ± 7.99 N for group A, 42.04 ± 19.68 N for group B, 28.69 ± 11.90 N for group C and 54.23 ± 7.41 N for group D. Means and standard deviations of the tensile failure load are shown in Table 1. Analysis of variance revealed statistically significant differences among studied groups (P < 0.001). Group D (Labial surface bevel + air abrasion) demonstrated highest mean tensile failure load followed by group B (labial surface bevel), group C (conventional + air abrasion) and the last group A. Compared to other groups, conventional group (A) demonstrated the lowest tensile failure load. A comparison of the mean values observed for all of the studied groups revealed statistically significant difference between group A and C (P = 0.003), groups A and B (P < 0.001), groups A and D (P < 0.001), groups B and C (P = 0.028), groups B and D (P = 0.027) and also groups C, D (P < 0.001) (Figure 2).

**Table 1:** Tensile failure load (Newton) of composite resin bonded to teeth

Groups	N	Mean	Std. Deviation	Minimum	Maximum
A (conventional)	25	20.66	7.99	10.35	43.85
B (labial surface bevel)	25	42.04	19.68	13.35	72.80
C (conventional + air abrasion)	25	28.69	11.90	8.17	58.00
D (labial surface bevel + air abrasion)	25	54.23	7.41	40.35	74.00
Total	100	36.41	17.97	8.17	74.00

## Discussion

The esthetic restoration of anterior primary teeth can be quite challenging not only because of the available materials and techniques, but also from the patients’ and parents’ point of view. Although current evidence indicates various novel techniques for restoring carious lesions in the anterior primary teeth, these restorations have been known to have less retention compared to the same in the permanent dentition. In many instance, the retention of class III restorations is not adequate because not enough surface area of the tooth was etched and bonded2. Therefore, the present study aimed to determine how different preparation techniques with and without air abrasion treatment influence the tensile failure load of class III composite restorations in primary teeth.

The overall direction of the results is supportive of the notion that air abrasion in conjunction with modified preparation results in a significant increase in tensile failure load. The highest bond strength was attained in the samples that were prepared using labial surface bevel treated with air abrasion, followed by labial surface bevel alone, conventional preparation treated with air abrasion and conventional preparation alone. Samples in group D demonstrated 50% more tensile failure load compared to group A, 38% increase compared to group C and 18% increase compared to group B.

The improved bond strength found in this study can be attributed the ability of air-abrasion in creating a rough surface for increasing micromechanical interlocking, surface energy, wettability and the bond area induced by labial surface bevel, using aluminum oxide particles. In addition, our findings showed significant increase in tensile failure load in group C compared to that in group A and also in group D compared to group B. Thus, it could be inferred that surface preparations along with air abrasion used in the samples of group D have likely resulted in a substantial increase of the surface energy. Surface energy can be used to develop wettability envelops to predict wetting of substrate by the adhesive. Wetting is required for good bond and intimate contact between tooth and restoration.^19^ Thus, it seems logical to assume that air abrasion produces increased surface area which would then improve the effectiveness of etching by increasing the wettability of the enamel.^20^ However, some studies showed that air abrasion is not an acceptable replacement for etching prior to bonding, and that air abrasion alone without acid etching does not increase the bond strength.^21,22^

Furthermore, the present study confirms previous findings that the use of Al_2_O_3_ air-abrasion followed by the application of phosphate monomer-based primers or resin cement produces more reliable results.^23-27^ Some studies have reported high surface roughness and formation of longer tags with Al_2_O_3_ particles.^28-30^ Increasing the surface roughness and bonding surface area leads to improved wetting behavior of adhesives.^19,31-33^, Air abrasion with Al_2_O_3_ particles is the surface treatment that causes micro-retentive features.^34^ In the morphological analysis of the enamel surface, Katora et al^35^ observed that the presence of superficial irregularities altered the surface of the enamel when Al_2_O_3_ particles were applied. In a similar line, Costa et al^30^ found that Al_2_O_3_ particles produced a significantly high surface roughness compared to no surface treatment and roughening with a diamond bur. Roughening the substrate surface due to phase transformation by the higher impact energy of particles promotes adhesion, since it allows the resin composite to flow into the surface and form irregularities on the substrate surface.^36-38^This increase in surface roughness may be one explanation for the higher tensile failure load in samples of group C compared to that of group A and group D compared to group B. This surface roughness most likely lends itself to an increase in micromechanical retention. These highly irregular surfaces may provide a suitable surface for good adhesion to composite resin as reported in the previous studies using air abrasion.^39^ However, this result is in contrast with some findings suggesting a decrease in resin bond strength in air abrasion treated surfaces due to the increased capability of acid to over-demineralise the dentin surface, causing collagen collapse and the deposition of calcium phosphate, which disrupts penetration of the adhesive.^9,40^ In addition, it has been suggested that Al_2_O_3_ air-abrasion (50 μm) along with acid etching associated with dentin surface alterations caused no increase in bond strength.^41-43^ While the reason for this discrepancy is not clear, it may be related to abrasion variables such as particle size or pressure. On the other hand, the difference in increase of tensile failure load in group C compared to group A was 12%, while the difference between groups D and B was 18%. This difference can be related to the larger bonding area due to modified preparation in group D compared to group C, therefore air abrasion particles affected more surface area.

In comparison of conventional + air abrasion (group C) and labial surface reduction (group B), group B showed a higher tensile failure load. The superior results of labial surface bevel preparation are probably related to increased surface area achieved by this procedure compared to air abrasion without labial bevel. The increasing of tensile failure load was 20% in group B compared to group C.

In addition, significantly higher bond strength in group B compared to that in group A and also in group D compared to that of group C can be attributed to the larger bonding surface area for micromechanical retention. Although consistent with our findings, Piyapinyo and White^28^showed that modified Cl III preparation in primary teeth had significantly higher mean failure load than the conventional preaparation, this makes the tooth more vulnerable to microleakage and marginal failure due to the larger surface involvement.To overcome this problem we used air abrasion in our study based on the results of the previous studies that concluded addition of air abrasion for treatment of preparations resulted in a gap-free adaptation between composites and dentin in most cases.^13,28^

Therefore, based on the results of the present study, it can be concluded that pretreatment with air abrasion in cavity preparation may cause more retention of composite resin in anterior primary teeth. This improved retention of Cl III composite restorations may be attributed to the increased surface roughness, bond area, surface energy, and wettability. The extent to which the results of the current investigation may be extrapolated for the clinical scenario and how it may affect clinical retention of Cl III composite restorations in anterior primary teeth is yet to be addressed.
